# Supplementary figures and images for: Molecular Detection of Porcine Parvovirus 5 in Domestic Pigs in Russia and Propagation of Field Isolates in Primary Porcine Testicular Cells
Source: Vet Sci. 2025 Jun 1;12(6):535. doi: 10.3390/vetsci12060535 (PMC12197641; doi:10.3390/vetsci12060535)

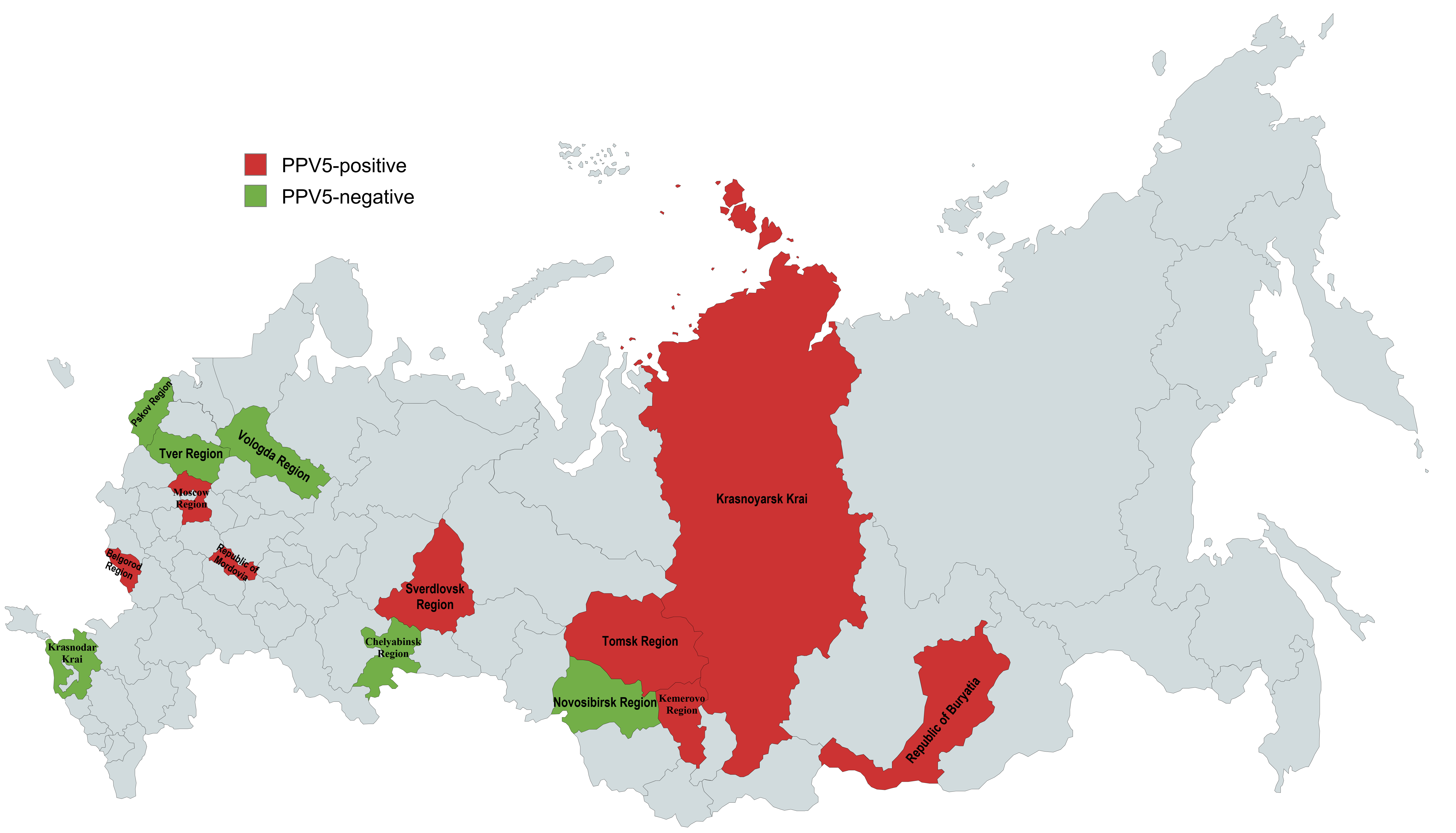

Supplement: Supplementary file 1 [file vetsci-12-00535-s001.zip › Supplementary_Figure_S1.png]
